# Factors influencing Patients’ Utilization of Dental Health Services in Jazan, Kingdom of Saudi Arabia

**DOI:** 10.5005/jp-journals-10005-1479

**Published:** 2017-02-01

**Authors:** Faeq A Quadri, Fatimahi AM Jafari, Alanood TS Albeshri, Abdulaziz M Zailai

**Affiliations:** 1 Assistant Professor and Course Coordinator, Department of Preventive Dentistry, Jazan University, Jazan Kingdom of Saudi Arabia; 2 Dentist, Department of Preventive Dentistry, Jazan University, Jazan Kingdom of Saudi Arabia; 3 Dentist, Department of Preventive Dentistry, Jazan University, Jazan Kingdom of Saudi Arabia; 4 Consultant Endodontist, Department of Endodontics, Ministry of Health, Jazan, Kingdom of Saudi Arabia

**Keywords:** Health services, Jazan, Kingdom of Saudi Arabia, Oral health.

## Abstract

**Introduction:**

One way of prevention and early detection of oral diseases is by utilizing the dental health care services on a regular basis. The current study aims to know the factors that play a role in influencing the dental service utilization in Jazan, Kingdom of Saudi Arabia.

**Materials and methods:**

A cross-sectional survey using a self-administered questionnaire was designed and implemented. Study subjects comprised of patients visiting the dental clinics at Jazan University and the primary dental centers of five different suburbs in Jazan region of Kingdom of Saudi Arabia. Items in the questionnaire were grouped into three sections; “demographic details,” “self-reported dental visits,” and “potential factors” contributing to dental visits. Chi-square p-value of 0.05 or less was considered as significant and logistic regression with 95% confidence interval (CI) was performed to get more precise results.

**Results:**

The sample size was 395 (N) of which 44.8% were males and 53.4% were females. Less than half (45.8%) of the studied sample reported that their last visit to a dentist was within a span of one year and 33% of them think that a dentist should only be visited if they experience pain. Patients following instructions given by a dentist were 7 times [odds ratio (OR) = 0.13; CI = 0.04, 0.40] less likely to miss their regular dental appointments. Following this, patients receiving knowledge on their dental problems were seen to be twice (OR = 0.50; CI = 0.25, 0.98) less likely to be irregular with their dental visits. Finally, the patients who are better educated and literate were also 2 times (OR = 2.21; CI = 1.14, 4.28) more likely to be regular with their dental appointments in comparison with the patients who completed just their primary level education.

**Conclusion:**

Findings of this study will facilitate future oral health prevention programs to be more focused, thereby reducing the gap between high and low educated sectors of the population residing in Jazan.

**How to cite this article:** Quadri FA, Jafari FAM, Albeshri ATS, Zailai AM. Factors influencing Patients’ Utilization of Dental Health Services in Jazan, Kingdom of Saudi Arabia. Int J Clin Pediatr Dent 2018;11(1):29-33.

## INTRODUCTION

Oral health is a crucial and contributory factor to general health among people of any age group, but is often overlooked. One decisive way of prevention and early detection of oral diseases is by utilizing the dental health care services on a regular basis.^1^ The term “utilization” can be put forth as the actual attendance by individuals to an oral health care facility. Studies show that this oral health service utilization by general population can be influenced by various factors, such as cost of treatment, accessibility, waiting period, level of education, and presence of severe symptoms, such as pain.^1^ Few other contributing factors to forgo professional dental visits are poor perception of the importance of oral health, lack of knowledge on the provided dental services,^2^ and psychosocial and self-perceptional factors.^3,4^ It is also reported that these factors leading to minimum access or low dental care utilization is seen to affect not only individuals or communities but the nation as a whole, thus remaining to be a major public health challenge.^5^

Oral health care facilities in Kingdom of Saudi Arabia are provided by 2,408 primary dental practices and specialized centers along with dental clinics associated with universities across the country.^6,7^ Regardless of free treatment benefits provided by the government, only a small percentage of population visits the dental centers regularly.^8^ This indicates that there could be underlying potential barriers affecting the service utilization. One recent study conducted in Riyadh reported that patient satisfaction, education, employment, and number of members in the family were associated with dental service utilization.^8^ Apart from this, there are very few studies conducted to know about the barriers of dental service utilization in Kingdom of Saudi Arabia and till date, there are no such data available from the Jazan region. Hence, the current study aims to know the factors that play a role in influencing the dental service utilization in Jazan, Kingdom of Saudi Arabia.

## MATERIALS AND METHODS

A cross-sectional survey using a self-administered questionnaire was designed and implemented in the months from January 2017 till February 2017. Study subjects comprised of patients visiting the dental clinics at Jazan University and the primary dental centers of five different suburbs in the Jazan region of Kingdom of Saudi Arabia. Ethical clearance from the Research Board at College of Dentistry as well as from the Ministry of Health, Jazan was obtained. Adults who could answer the questionnaire without assistance were included in the study. Subjects who were physically or mentally challenged and who did not give consent to participate were excluded.

The questionnaire was designed by a team of dental professionals in English after a thorough literature review which was later translated into Arabic. Translated questionnaire was subjected to a series of validation steps as follows: two bilingual dental professionals assisted in performing the translation and reverse translation respectively. Understanding the concept of each item was given importance while translation and not just the literal meaning. Intraclass correlation of the questionnaire was performed on a pilot sample of 20 subjects, and a value of 0.87 was obtained.^9^ Items in the survey questionnaire were grouped into three sections; “demographic details,” “self-reported dental visits,” and “potential factors” contributing to dental visits.

Data were stored and analyzed using Statistical Package for the Social Sciences version 20 (IBM, USA). Descriptive analysis was reported in percentages and chi-square analysis was conducted in order to check the relation between the dependent variable (dental visits) and independent variables, i.e., education level and other perceived barriers. A p-value of 0.05 or less was considered as significant and logistic regression with 95% CI was performed to get more precise results.

## RESULTS

The sample size was 395 (N) during the mentioned study period with 100% response rate, of which 44.8% were males and 55.2% were females. Most of them were in the age group of 15 to 30 years with 42% reported to be tertiary educated. In the descriptive analysis of the assessed variables, it is observed that less than half (45.8%) of the studied sample reported that their last visit to a dentist was within a span of 1 year and 33% of them think that a dentist should only be visited if they experience pain (Table 1).

The percentage of patients who said that they would follow the instructions given by their dentist was very high (88%). About 68.6% of the patients said that their dentist listens and gives a detailed description on their oral health problems; but, nearly 37.7% were affirmative about their previous bad experience with a dentist. On asking if the dental practice usually visited is well equipped, only 24% responded positively. Oral health prevention programs or camps were never attended by 83.2% of the sample. An open-ended question was also put forth to report on qualitative analysis of the self-perceived barriers among this sample. The responses were later categorized according to the most common answers illustrated by the subjects. It revealed that delay in appointment (53.2%), absence of pain (16.8%), and fear of dentist (13%) were the top three self-reported barriers (Table 1).

**Table 1:** **Table 1:** Descriptive values of the assessed variables

*Variables*		*%*	
*Age (years)*			
Below 15		6.4	
15-30		49.9	
Above 30		43.7	
*Gender*			
Male		44.8	
Female		55.2	
*Level of education^3^*			
Primary		19.5	
Secondary		30.6	
Tertiary		42.0	
*Region*			
Jazan		29.1	
Sabya		19.2	
Abu Areesh		14.7	
Samtah		5.8	
Al Ardah		3.5	
*Dental visits*			
Every 6 months to 1 year		49.5	
More than 1 year		16.2	
When in pain		33	
Other reason		0.3	
*Dentists’ instructions*			
Yes		88	
No		12	
*Dentists’ explaining oral health problem*			
Yes		68.6	
No		31.4	
*Previous dental visits*			
Regular		54.2	
Irregular		45.8	
*Dental pain is the reason for current visit*			
Yes		38.8	
No		61.2	
*Patients attending oral health camps*			
Yes		16.8	
No		83.2	
*Previous bad experience with the dentist*			
Yes		37.7	
No		62.3	
*Availability of equipments in dental practice*			
Yes		24	
No		36	
I do not know		30	
No response		10	
*Qualitative response on self-perceived*			
*barriers by the patients^b^*			
Delay in appointment		53.2	
Absence of pain		16.8	
Fear of dentists		13	
Regular visit is not necessary		7	
Other reasons		10	

The total sample size was 395 (N); ^a^7.8% respondents did not mention about their level of education; ^b^It was an open-ended question where the top responses have been grouped together

**Table 2:** **Table 2:** Patients’ dental visits in relation to their education level

				*Dental visits*							
		*Regular^a^*		*Irregular*							
*Level of education*		*Once every 6 months or 1 year*		*More than 1 year*		*When in pain*		*Other reasons*		p-value*	
Primary		17		14		28		1		0.00	
Secondary		47		19		31		0			
Tertiary		87		13		37		0			

*Chi-square test, considering p-value ≤0.05 as significant; ^a^Patients’ visits of every 6 months or 1 year have been considered as “Regular visits”

**Table 3:** **Table 3:** Patients’ dental visits in relation to the perceived barriers

						*Patients’ perception of dental visits*					
		*Regular^a^*		*Irregular*							
*Barriers*		*Once every 6 months to 1 year*		*More than 1 year*		*When in pain*		*Other reasons*		*p-value*	
Delay in appointment		90		26		46		1		0.81	
No pain		89		22		39		0		0.00*	
No personal motivation		4		3		6		0		0.00*	
Fear of dentist		16		9		16		0		0.74	
Not equipped		69		23		47		0		0.32	
Bad experience		59		17		43		0		0.00*	
Oral promotion programs		28		0		10		1		0.01*	

*Chi-square test, considering p-value of ≤0.05 as significant; ^a^Patients’ visits of every 6 months or 1 year have been considered as “Regular visits”

**Table 4:** **Table 4:** Logistic model demonstrating the factors affecting patients’ regular dental visits

								*Confidence interval (95%)*			
*Factors*		*B*		*p-value*		*Adjusted OR*		*Lower*		*Upper*	
Dentists’ instructions^a^		2.03		0.00		0.13		0.04		0.40	
Explanation of dental problems^b^		0.69		0.04		0.50		0.25		0.98	
Attending oral health camps^c^		0.99		0.00		0.99		0.38		2.56	
Level of education^d^		0.79		0.01		2.21		1.14		4.28	

Binary logistic regression was performed categorizing visits of once every 6 months or 1 year as “Regular” and rest as irregular dental visits; ^a^Patients not following dental instructions; ^b^Dental problems not explained; ^c^Not attending oral health camps; ^d^Primary education; Age and gender were kept as covariates

Chi-squared analysis revealed that the level of education (p = 0.00) was significantly associated with the patients’ dental visits (Table 2). Visiting only if there is pain (p = 0.00), lack of self-motivation for a regular dental visit (p = 0.00), not attending an oral health camp (p = 0.01), or having a history of bad experience with the dentist (p = 0.00) were seen to be related to the patients’ regular dental visits (Table 3).

Logistic regression was performed with age and gender as covariates; and it portrayed that the patients’ following instructions given by a dentist were 7 times (OR = 0.13; CI = 0.04, 0.40) less likely to miss their regular dental appointments. Following this, patients receiving knowledge on their dental problems were seen to be twice (OR = 0.50; CI = 0.25, 0.98) less likely to be irregular with their dental visits. Finally, the patients who are better educated and literate were also 2 times (OR = 2.21; CI = 1.14, 4.28) more likely to be regular with their dental appointments in comparison with the patients who completed just their primary-level education (Table 4).

## DISCUSSION

Oral diseases, such as dental caries^10-12^ and oral cancer^13^ are prevalent in the population residing in the Jazan region of Kingdom of Saudi Arabia. There has been a significant advancement in dental technology and the scientific understanding of oral diseases, but one cannot deny the fact that all of this can only benefit an individual or a population if there is an access or utilization of available dental services. The current study was conducted to know the barriers that significantly influence the dental service utilization of patients visiting the dental clinics in the Jazan region of Kingdom of Saudi Arabia. The dependent variable assessed was self-reported dental visits, which was categorized into regular and irregular.

One difficulty faced by clinicians and also the academicians is to come to a conclusion as to how much is the time gap between each regular dental visit. Although many clinicians recommend 6-monthly visit, it has been questioned 25 years ago and remains a matter of discussion till today based on low-quality scientific evidence.^14^ Some professionals say that the period between check-ups should be based on individual patients’ risk.^15^ Yet another research portrays 1-yearly visit to a dentist as the most commonly used index to measure dental service utilization.^16^ In this study, based on the consensus and the usual practices in many countries, we have categorized dental care utilization as “regular” only if an individual in the population visits or has a self-perception of visiting a dental health care provider in a span of 6 months or 1 year.

The current study showed that the level of education of an individual is a significant contributing factor to dental service utilization and this is in accordance with a recent study conducted among another set of Arab population.^17^ Logistic regression also revealed that patients who had a higher education level were twice more likely to be regular in their oral health care visits. This indicates that the people with less education should be targeted when designing any oral health prevention program so that maximum benefit is derived. Current research also shows that a major chunk of the studied sample believed that an oral health care provider should be visited only if there is an existing pain; and this type of finding was reported by Ayo-Yusuf and Naidoo^18^ in their research. One more study conducted in a developed nation also concluded that people visited the dentist for pain relief rather than any preventive care.^19^

This research also portrays that a past bad experience with an oral health care provider is also a significant barrier to dental service utilization and this is similar to a study conducted in United Kingdom wherein the bad experience was specifically related to pain during treatment.^20^ The largest contributing factor according to the current survey was “dental instructions”; and it showed that the individuals who followed instructions given by a dentist were 7 times more likely to be regular in their oral health care visits. This is in line with another research which described that, if the dentist’s and their team genuinely listen to the concerns of their patients, then they would follow the instructions which in turn leads to regular utilization of the oral health services.^21^

It is to be noted that there is no previous evidence of this kind from the Jazan region of Kingdom of Saudi Arabia, and thus, the information provided is of high value. But the authors would also like to display the following limitations to the findings obtained. The study may have been subjected to reporting bias on the part of the respondents. Cause-and-effect relationship between the dependent variable (perceived dental visits) and the independent variables (barriers) is not feasible due to its cross-sectional design.

The authors would like to conclude by commenting that, though Kingdom of Saudi Arabia provides free primary oral health services to its citizens, there exist certain barriers to its utilization. It goes without a doubt that the findings of this study will facilitate future oral health prevention programs to be more focused, thereby reducing the gap between high and low educated sectors of the population residing in Jazan.
